# Assessment of Hydrogels for Intra-Articulate Application, Based on Sodium Hyaluronate Doped with Synthetic Polymers and Incorporated with Diclofenac Sodium

**DOI:** 10.3390/ijms26157631

**Published:** 2025-08-06

**Authors:** Dorota Wójcik-Pastuszka, Maja Grabara, Witold Musiał

**Affiliations:** Department of Physical Chemistry and Biophysics, Faculty of Pharmacy, Wroclaw Medical University, ul. Borowska 211A, 55-556 Wrocław, Poland; dorota.wojcik-pastuszka@umw.edu.pl (D.W.-P.); majagrabara00@gmail.com (M.G.)

**Keywords:** diclofenac sodium, sodium hyaluronate, intra-articular hydrogel, viscosity study, release kinetics

## Abstract

The intra-articular application of drugs has gained considerable interest with regard to formulations for advanced drug delivery systems. It has been identified as a potential route for local drug delivery. A drug agent is usually incorporated into the hydrogel to prolong and control the drug release. This study aimed to design and evaluate an intra-articular hydrogel based sodium hyaluronate, which was modified with an additional polymer to enable the sustained release of the incorporated anti-inflammatory agent, diclofenac sodium (NaDic). Viscosity studies, drug release tests and FTIR−ATR measurements, as well as DSC analysis, were carried out to evaluate the obtained formulations. The viscosity measurements were performed using a rotational viscometer. The drug release was carried out by employing the apparatus paddle over the disk. The concentration of the released drug was obtained spectrophotometrically. The results revealed that the addition of the second polymer to the matrix influenced the dynamic viscosity of the hydrogels. The highest viscosity of (25.33 ± 0.55) × 10^3^ cP was observed when polyacrylic acid (PA) was doped in the formulation. This was due to the hydrogen bond formation between both polymers. The FTIR−ATR investigations and DSC study revealed the hydrogen bond formation between the drug and both polymers. The drug was released the slowest from hydrogel doped with PA and 17.2 ± 3.7% of NaDic was transported to the acceptor fluid within 8 h. The hydrogel based on hyaluronan sodium doped with PA and containing NaDic is a promising formulation for the prolonged and controlled intra-articulate drug delivery of anti-inflammatory agents.

## 1. Introduction

The inflammation of synovial articulations is a pathological process affecting one or more joints. It causes pain, swelling, redness, stiffness, and limited mobility. The condition can be acute or chronic and may arise from various reasons. [1,2]. Synovial articulation diseases are characterized by inflammation of the synovial membrane of the joints and may lead to the degeneration of joint cartilage [3,4]. The joint cavity is filled with synovial fluid, which provides lubrication and nutrition to the joint cartilage. Under physiological conditions, synovial fluid is clear, viscous, and contains a small number of cells (up to 200 cells/µL). Its pH ranges from 7.2 to 7.4. However, it changes in inflammatory diseases such as rheumatoid or infectious arthritis. The number of nucleated cells increases, often exceeding 8000 cells/mg [5]. In bacterial joint infections, a significant increase in the percentage of neutrophils is observed [6]. In inflammatory conditions, the pH of the synovial fluid decreases below physiological values (7.2–7.4), as a result of the inflammatory process and infection [7,8,9]. The concentration of protein in inflamed synovial fluid may increase, reflecting the severity of the inflammatory process [10]. In addition, the glucose concentration in inflamed synovial fluid may be reduced compared to plasma [11].

**Figure 1:**
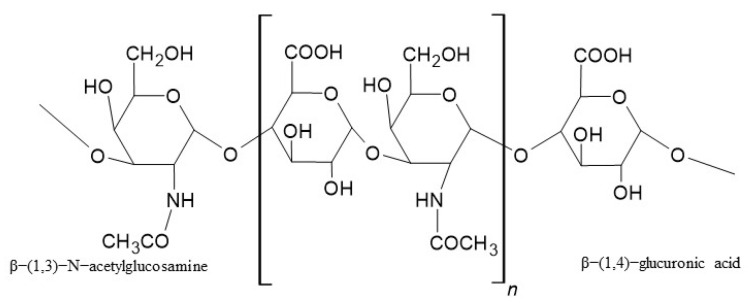
The structure of HA.

An important component of the synovial fluid is hyaluronic acid (HA), which is responsible for its viscosity and lubricating properties, ensuring proper joint function. HA is a linear, negatively charged, water-soluble, biodegradable, and viscoelastic biopolymer. Its structure (Figure 1) consists of the two repeating units β−(1,3)−N−acetylglucosamine and β−(1,4)−glucuronic acid, linked by β−1,3−and β−1,4−glycosidic bonds. The great flexibility and solubility of HA polymers arise from the β−1,3 connections [12].

In inflammatory joint conditions, significant changes occur in HA metabolism, which affects its structure and function. Under physiological conditions, the HA in synovial fluid is characterized by a high molecular weight of up to 7 million Daltons, which ensures the appropriate viscosity and elasticity of the fluid. One of the most important properties of HA is its ability to absorb large amounts of water. High-molecular-mass HA aqueous solutions are cohesive, viscous, and lubricating. HA forms hydrogen bonds both within one macromolecule, and between the adjacent molecules. It should be noted that the aqueous molecule can act as a bridge between the two connected functional groups of HA. This primary structure, along with hydrogen bonding, contributes to the formation of secondary and tertiary structures. Finally, the biopolymer in solution may adopt a left-handed single or double helical conformation [13]. This helical configuration of HA traps water, increasing its weight approximately 1000 times. Additionally, the mesh structure of HA hydrogel acts as a barrier against exogenous substances, including bacteria and other infectious agents [14].

In inflammatory conditions, such as rheumatoid arthritis, a decrease in both the molecular weight and concentration of HA in synovial fluid is observed. These changes lead to a decrease in the viscosity of synovial fluid, which can result in the impairment of joint cushioning and lubrication, increasing pain symptoms and limiting mobility [15,16]. Inflammation stimulates the activity of enzymes such as hyaluronidases and reactive oxygen species, which degrade HA. The increased activity of these enzymes leads to the fragmentation of HA, resulting in the formation of chains with a lower molecular weight. These smaller fragments may exhibit pro-inflammatory properties, thereby intensifying the inflammatory response in the joint [17]. In response to the degradation of endogenous HA in inflammatory conditions, therapy that involves the intra-articular administration of exogenous high-molecular-weight hyaluronic acid is used. The aim of this therapy is to restore the rheological properties of synovial fluid, reduce pain, and improve joint function [18,19]. However, HA has a short retention time in the joint cavity. To obtain effective therapy, intra-articular injections should be repeated. Multiple intra-articular administrations are inconvenient for patients; therefore, studies are focused on increasing the retention of HA in the synovial fluid by combining it with a second polymeric molecule [20]. Synthetic polymers such as polyacrylic acid (PA), ammonium acryloyldimethyltaurate/VP copolymer (Aristoflex AVC, AX), polyvinyl acetate-polyvinylpyrrolidone mixture (PVA−PVP, kollidon SR) and polyethylene glycol (PEG) are commonly used in pharmaceutical technology. PA is a biocompatible, non-toxic, biodegradable polymer and therefore is widely used as a component of drugs and prolonged-release gels [21]. Additionally, the PA concentration that was proposed as a gelling agent in the antioxidant gel was in the range of 0.5−2.5% [22]. AX is used in pharmacy and cosmetology as a gelling agent in gels, creams, and serums. It was found that AX improved the chemical and physical properties of enamel [23]. This polymer was also proposed as vaginal gel [24]. The typical level of AX in hydrogels recommended by the manufacturer is in the range of 0.5−2.0% [25], and the amount of AX in hydrogels for aging skin and for treating blepharitis and demodicosis suggested by Baranova et al. [26] was between 0.5 and 1.5%. PVA and PVP macromolecules were proposed for biomedical applications based on the synergistic effect of mixtures between PVA and PVP. PVA possesses properties such as solubility in water, diffusivity, crystallinity, biodegradability, adhesion, and mechanical strength, which are responsible for its applicability in the medical field. Moreover, a preclinical and clinical study indicated that PVA hydrogels have high in vivo biocompatibility, a low level of oral toxicity, and an absence of mutagenic activity [27]. PVP shows good environmental stability, bio- and hemocompatibility, biodegradability and low cytotoxicity. This polymer has been accepted by the US Food and Drug Administration as a safe polymer for biological experiments, and is popular in the pharmaceutical and biomedical field [28]. PEG 4000 is a tasteless, non-toxic, water-soluble, and non-absorbable polymer. It is not metabolized by colonic bacteria and, for this reason, it is safe and effective in the treatment of constipation in children [29]. Additionally, Turecek et al. [30] revealed that, in mammalian organisms, there are no enzymes that are able to metabolize PEG; this explains the lack of toxicity of this macromolecule in humans.

The most common medications for the temporary relief of joint diseases are nonsteroidal anti-inflammatory drugs (NSAIDs), corticosteroids and anti-rheumatic agents [31]. One of the NSAIDs most widely prescribed for the treatment of pain and inflammation, including joint pain, is diclofenac sodium (NaDic). It is used as a primary treatment for rheumatoid arthritis, osteoarthritis (OA), ankylosing spondylitis (AS), and gout. This medication helps relieve joint pain and stiffness [3,4,32,33,34].

**Figure 2:**
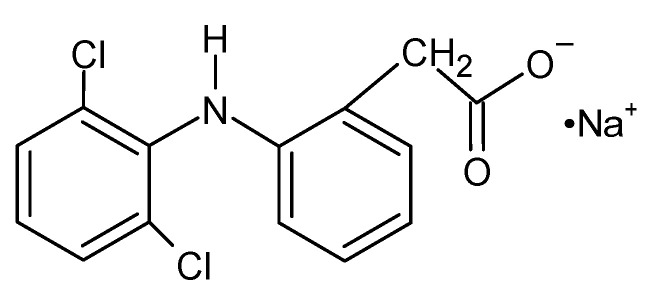
The chemical structure of NaDic.

NaDic is available on the market in various forms for local, oral, and parenteral use. These include immediate- and prolonged-release tablets, rectal suppositories, injectable solutions, topical gels and ointments, as well as eye drops [35]. Francio et al. [36] reported that NSAIDs, such as NaDic, are generally well tolerated. However, studies have shown that local administration is better tolerated than oral administration, as it helps to avoid gastrointestinal side effects. When applied locally, NaDic penetrates the skin and synovial tissues, entering the synovial fluid where it accumulates in inflamed areas. This reduces the levels of prostaglandin E2 (PGE2) and other pro-inflammatory biomarkers [4]. The chemical structure of NaDic is presented in Figure 2. The substitution of two chlorine (Cl) atoms into the phenyl ring of NaDic enhances the drug molecule’s ability to fit into the substrate-binding pocket of the COX enzyme [37,38]. NaDic’s mechanism of action involves the inhibition of the cyclooxygenase (COX) enzyme, which is crucial for prostaglandin synthesis—key mediators in the development of inflammation and pain. [39].

NaDic, which is quickly metabolized, is applied in large doses, which may cause digestive problems (side effects), especially in elderly people. Local administration overcomes these inconveniences and appropriate polymers, forming a spatial network, ensure constant and prolonged drug release [33]. A hydrogel film composed of chitosan (CS) and polyvinylpyrrolidone (PVP), with epichlorohydrin as a cross-linking agent, was proposed as a carrier for NaDic, enabling controlled drug release [40]. Suhail et al. [41] proposed carbomer-based formulations for the prolonged and controlled release of NaDic. Li et al. [42] developed a HA-based hydrogel containing NaDic for wound healing and Küçüktürkmen et al. [43] investigated an in situ gelling intra-articular delivery system of a hydrogel containing NaDic-loaded nanoparticles.

The combination of an HA-based drug carrier, which possesses anti-inflammatory properties, with NaDic as an anti-inflammatory drug may be an effective therapeutic strategy for the treatment of synovitis.

The aim of this study was to develop novel hydrogel compositions of NaDic, based on HA or HA combined with an auxiliary polymer such as PA (polyacrylic acid), AX (ammonium acryloyldimethyltaurate/VP copolymer), PVA−PVP (polyvinyl acetate-polyvinylpyrrolidone mixture) or PEG (polyethylene glycol). The designed hydrogels were used to ensure the controlled prolongation of NaDic release. This investigation also used drug dissolution to analyze the amount of the drug released and determined the release kinetics of the drug from the formulations to understand the mechanism of drug transport from the carrier into the acceptor fluid. An investigation of the viscosity of the preparations and FTIR and DSC studies were carried out to evaluate and analyze the potential interaction among the components of the compositions.

## 2. Results and Discussion

### 2.1. pH and Viscosity Investigation

The composition of the hydrogels is presented in Table 1. The pH values of the obtained hydrogels are shown in Table 2. It was noticed that the pH of formulations F1−F5 was higher in comparison to the respective pH values of hydrogels not containing the drug, which were 7.18 ± 0.10, 4.26 ± 0.03, 6.67 ± 0.01, 6.82 ± 0.08, and 7.15 ± 0.02 for F1′−F5′ NaDic free, respectively [44]. NaDic is a salt whose pKa is 4.35 [45]. According to USP [46], the pH of the 1% aqueous solution of NaDic is in the range 7.5–8.5. It underwent dissociation involving the detachment of a sodium ion, the subsequent binding of a hydrogen ion, and the release of a hydroxide ion. The formed OH− ions were responsible for the alkaline pH, and explained the fact that the incorporation of NaDic into the used carriers increased the pH values of these preparations.

**Figure 3:**
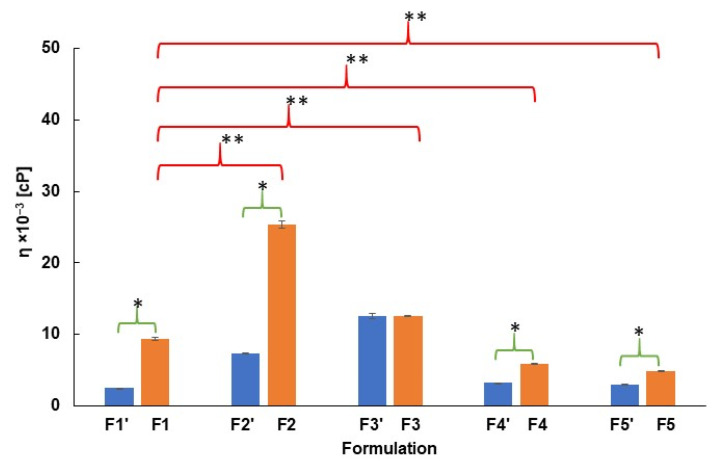
The dynamic viscosity of hydrogels F1−F5 (in yellow) and F1′−F5′ (in blue) at 37 °C [44]. One asterisk * indicates the differences arising from NaDic incorporation into hydrogels and two asterisks ** indicate the differences obtained from the addition of a second polymer to the hydrogels, *n* = 6.

The dynamic viscosity of formulations F1−F5 is presented in Figure 3. For comparison, the dynamic viscosity of carriers F1′−F5′, NaDic free, is also shown. The analysis of the influence of NaDic incorporation into the matrix on its viscosity using t-Student’s test indicated that, apart from the hydrogel F3, the addition of the drug increased the dynamic viscosity. These results may suggest an interaction between NaDic and the carrier. Giubertoni et al. [47] revealed that the amide and carboxylate groups coming from the HA molecule interact via an intrachain hydrogen bond: O=C–N–H···O–C=O–. NaDic possesses a carboxylate group in its structure (Figure 2) and suggests that the drug molecule can also form a similar hydrogen bond with the −NH− group belonging to the HA chain. Additionally, the −NH− group occurs in the NaDic molecule as well, suggesting that there is an interaction with the carboxylate group of HA. A study by Rodrigues et al. [48] indicated that diclofenac may also form intermolecular hydrogen bonds between the oxygen atom from the carbonyl group and the H atom belonging to the OH group. In aqueous solutions, hydrogen bonds may occur between diclofenac and water molecules. The strength of the interaction varies, and sometimes may be close to a covalent bond.

The increase in the dynamic viscosity of hydrogels F2, F4 and F5 arising from the introduction of NaDic into the preparation may also be related to the interaction of the drug with an additional polymer such as PA, PVA−PVP, PEG, respectively. The PA chain contains a carboxyl group in each monomer unit [49]. This may suggest that there is an interaction between these groups and the −NH− groups present in NaDic molecules. It was revealed that PVP carbonyl groups may interact with amine −NH− groups as well as with hydroxyl groups; these interactions predominantly occur via intermolecular hydrogen bonding and van der Waals interactions [50]. Additionally, Lewandowska et al. [51] postulated the interaction between the carbonyl group of PVP and the hydroxyl and carboxylate groups of HA. This led to the conclusion that PVP may interact with the −NH− groups belonging to NaDic, as well as with −COO− from the drug. Rahim et al. [52] found a bond formation between two −C=O groups, which may suggest that there is an interaction between PVA and NaDic, as well as between NaDic and PVP, and explain the increase in the dynamic viscosity of F4 in comparison to the drug-deprived carrier. In the case of formulation F5, the increase in the dynamic viscosity may be a result of a bond formation between the ether oxygen coming from PEG, and the hydroxy group of carboxylic acid in NaDic [53]. According to the following hydrolysis equations:

NaDic → Na^+^ + Dic^−^ (1)

Dic^−^ + H_2_O → DicH + OH^−^ (2)

in these conditions, NaDic possesses a −COOH group that is a good hydrogen bond donor. A similar interaction between PEG and HA in aqueous solution was found by Le−Deygen et al. [54]. The ether −C−O−C− group of PEG formed a bond with hydroxyls of the HA molecule presented by the −COOH group. This interaction was even more favorable than that between the −C−O−C− group of PEG with water.

The lack of changes in the dynamic viscosity of the F3 formulation after the incorporation of the drug into the drug-free hydrogel F3′ may be explained by the absence of an interaction between NaDic and both polymers, although the bond formation between HA and AX was claimed [44].

The discrepancies in the dynamic viscosity of the hydrogels arising from the incorporation of the auxiliary polymer was investigated using t-Student’s test and formulation F1, not containing the second polymer, was established as the reference sample. The obtained differences are marked in Figure 3. It was found that the addition of PA or AX to the HA matrix increased the dynamic viscosity of hydrogels F2 and F3 in comparison to the dynamic viscosity of F1. HA and PA are capable of creating hydrogen bonds and forming compositions with a high viscosity through spatial interactions and the retention of each other in the hydrophilic matrix [44]. Jimenez et al. [55] reported that the addition of PA as an auxiliary polymer—which similar to HA swells under the influence of water and creates a three-dimensional network—results in the mutual interpenetration of the two polymer phases, leading to an increase in the density and therefore to an increase in the viscosity of the entire system. A similar explanation may be proposed in the case of the increase in the viscosity of F3. HA develops its own gel network through hydrogen bonds and electrostatic interactions. The addition of AX leads to a synergistic increase in the viscosity of the resulting system, because the networks of both polymers interpenetrate, building a more compact structure. However, in the case of formulations F4 and F5, the dynamic viscosity decreased after introducing the PVA−PVP mixture and PEG, respectively. The decrease in viscosity is a result of the disruption of the HA network by PVP through system dilution and the competition for water molecules, which ultimately causes the loosening of the hydrogel structure. The interactions between HA and PVP weaken the physical cross-linking within the hydrogel and may lead to its destructuring. PVP is a hydrophilic polymer and can bind water that participates in the hydration of HA. This causes a decrease in HA swelling and finally reduces its viscosity [56]. This is consistent with the results obtained by Lewandowska et al. [51], which revealed that in certain conditions, the molecular interactions between HA and PVP hinder interactions between the polymer chain and solvent, weakening the impact of solvation and leading to a decrease in the dynamic viscosity. Ma et al. [57] revealed that PEG possesses the ability to loosen the three-dimensional structure of hydrogels. Additionally, PEG increased the osmotic pressure of fluids and PEG-induced osmotic stress reduced the amount of water molecules by reducing the hydration of HA, which ultimately reduces the viscosity of the hydrogel [58,59].

### 2.2. FTIR-ATR Study

**Figure 4:**
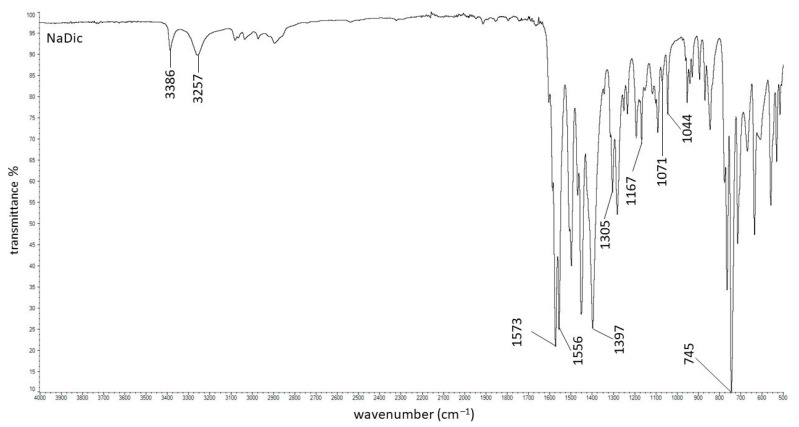
The FTIR−ATR spectrum of pure NaDic.

The FTIR−ATR spectrum of pure NaDic is presented in Figure 4. The characteristic bands at 3386 cm^−1^ and 3257 cm^−1^ were found and they were assigned to the NH stretching of the secondary amine and NH−O stretching vibrations, respectively. Strong signals at 1573 cm^−1^ attributed to the C=O stretching of the carboxylate vibrations (asymmetric) and at 1397 cm^−1^ (symmetric) were noticed. A peak at 1556 cm^−1^ corresponding to the C=C ring stretching and a strong signal at 745 cm^−1^ coming from C-Cl stretching were observed. This spectrum exhibited the ring breathing bands characteristic of aromatic rings at 1071 cm^−1^ and 1044 cm^−1^. The peaks at 1305 cm^−1^ and 1167 cm^−1^ resulting from C−N stretching were also found. All these bands corresponded with data in the literature [60,61,62].

**Figure 5:**
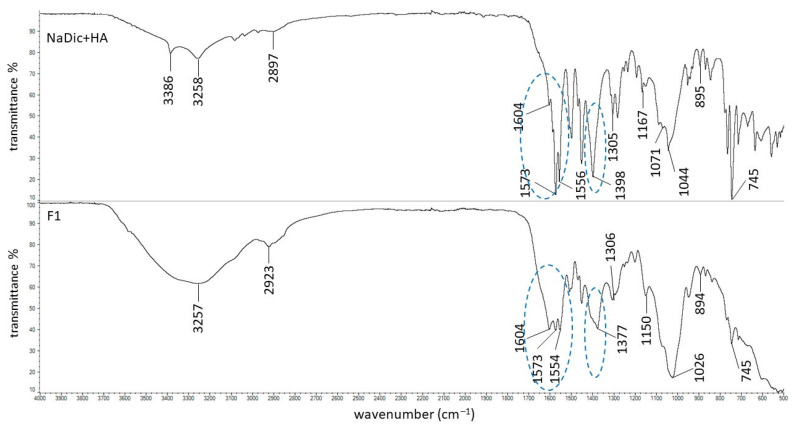
The FTIR−ATR spectra of the physical mixture of ingredients of formulation F1 and formulation F1; differences between the spectra are marked in blue oval.

**Figure 6:**
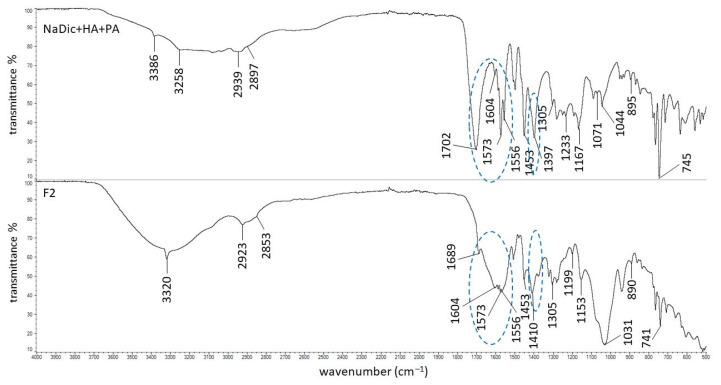
The FTIR−ATR spectra of the physical mixture of ingredients of formulation F2 and formulation F2; differences between the spectra are marked in blue.

**Figure 7:**
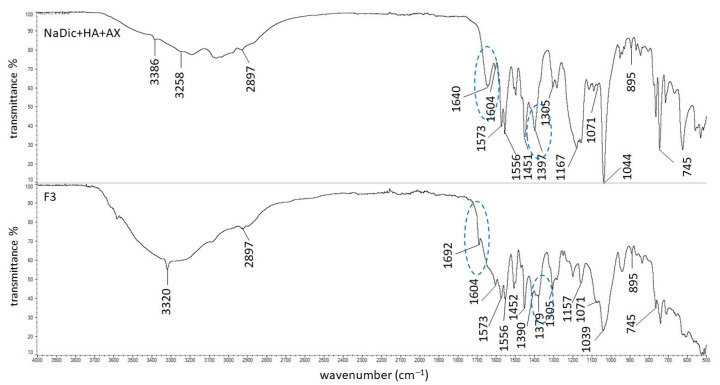
The FTIR−ATR spectra of the physical mixture of the ingredients of formulation F3 and formulation F3; differences between the spectra are marked in blue.

**Figure 8:**
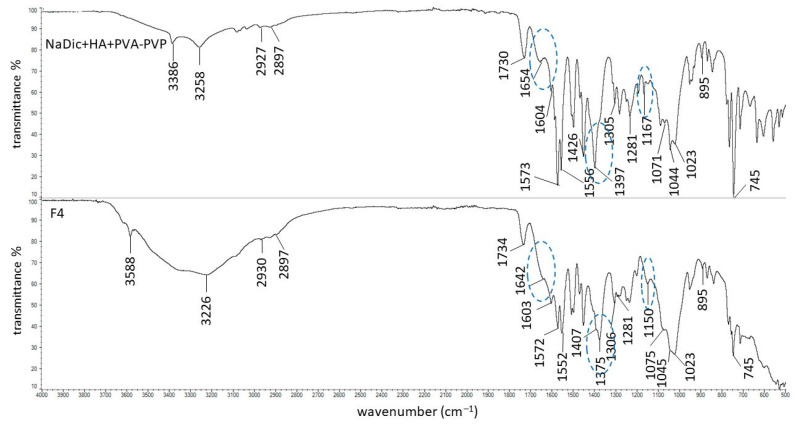
The FTIR−ATR spectra of the physical mixture of the ingredients of formulation F4 and formulation F4; differences between the spectra are marked in blue.

**Figure 9:**
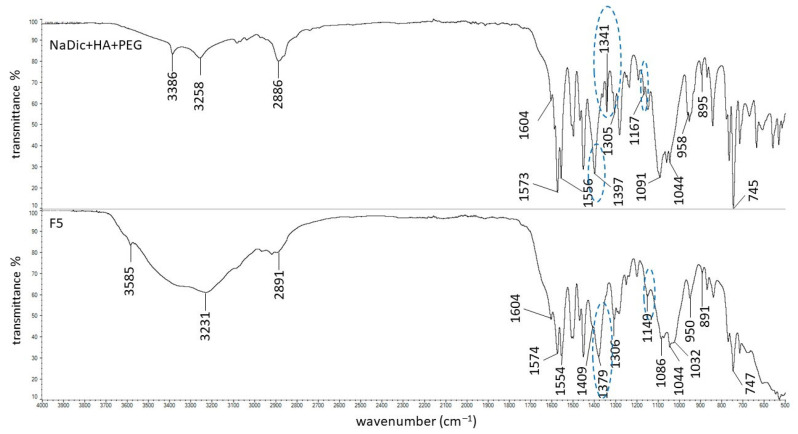
The FTIR−ATR spectra of the physical mixture of the ingredients of formulation F5 and formulation F5; differences between the spectra are marked in blue.

**Figure 10:**
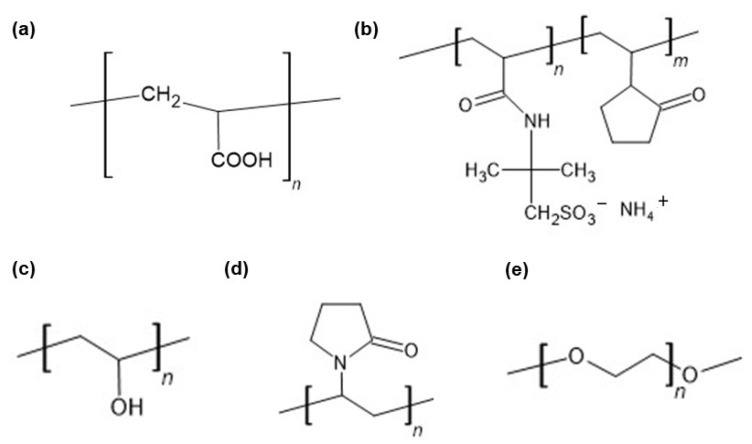
The chemical structures of (**a**) polyacrylic acid; PA (**b**) ammonium acryloyldimethyltaurate/VP copolymer; AX (**c**) polyvinyl acetate; PVA (**d**) polyvinylpyrrolidone; PVP (**e**) polyethylene glycol; PEG.

The FTIR−ATR spectra of pure polymers used in the present work have already been published. The main bands of HA were observed at 3285, 2897, 1604, 1407, 1376, 1032, and 895 cm^−1^. The characteristic peaks of PA were noticed at 2936, 1698, 1453, 1413, and 1220 cm^−1^ [63]. The signals of AX occurred at 1640, 1544, 1440, and 1388 cm^−1^. The FTIR−ATR spectrum of the PVA−PVP mixture showed the main maxima at 3434, 2937, 1730, 1654, 1426, 1291, and 1023 cm^−1^. The FTIR−ATR spectrum of PEG exhibited bands at 3449, 2882, 1097, 1341, and 958 cm^−1^ [44]. The FTIR−ATR spectra of the physical mixtures of ingredients comprising formulations F1−F5, as well as dried formulations F1−F5, are presented in Figure 5, Figure 6, Figure 7, Figure 8 and Figure 9. The chemical structures of the auxiliary polymers are shown in Figure 10.

The characteristic bands of the components of all formulations were present on the corresponding FTIR−ATR spectra of their physical mixtures. However, some peaks were invisible due to overlapping by neighboring bands. The HA signal at 3285 cm^−1^ was very broad and it was difficult to assign it precisely to the spectra presented in Figure 5, Figure 6, Figure 7, Figure 8 and Figure 9. The HA peaks at 1407 cm^−1^ and 1376 cm^−1^ were weak and were also invisible as the stronger signal coming from NaDic at 1397 cm^−1^ covered them. The HA band at 1032 cm^−1^ was probably present at 1044 cm^−1^. At this wavenumber, the band of NaDic occurred. However, its shape resembled the shape of a HA peak rather than NaDic, which was more sharp.

The signals on the spectrum of the physical mixture of NaDic, HA and PA (Figure 6) present at 1701 cm^−1^ and 1233 cm^−1^ corresponded to the peaks at 1698 cm^−1^ and 1220 cm^−1^ on the pure PA spectrum, respectively [63]. The weak peak of PA at 1413 cm^−1^ was not found on the spectrum of the NaDic, HA and PA physical mixture.

The signals of AX at 1544, 1440, and 1388 cm^−1^ on the spectrum of the physical mixture of NaDic, HA and AX (Figure 7) were overlapped by strong signals at 1556, 1451, and 1397 cm^−1^, respectively, which were attributed to NaDic [44].

The band of the PVA−PVP mixture at 3434 cm^−1^ was invisible on the spectrum of the physical mixture of NaDic, HA and PVA−PVP (Figure 8), probably because it was wide and weak and was overlapped by another signal. The bands at 1426 cm^−1^ and 1291 cm^−1^ were found at 1452 cm^−1^ and 1281 cm^−1^, respectively.

The signal of PEG at 3449 cm^−1^ on the spectrum of the physical mixture of F5 components (Figure 9) was not visible because it was very weak and broad on the spectrum of pure PEG [44]. The PEG band at 2882 cm^−1^ was slightly shifted and was observed at 2886 cm^−1^. This signal was overlapped by the HA peak at 2897 cm^−1^, in a manner similar to the PEG band expected at 1097 cm^−1^, which was observed at 1091 cm^−1^—possibly as a result of overlapping with the HA band at 1071 cm^−1^.

An FTIR−ATR study of formulations F1−F5 without the drug was carried out in previous work. The hydrogen bond formation between HA and water was observed. Additionally, it was suggested that there are interactions between HA and the polymers incorporated into the hydrogels [44]. The hereby presented study focused on the interactions between the drug and polymers included in the formulations. The spectrum of formulation F1 is shown in Figure 5. It was interesting that the band of NaDic at 3257 cm^−1^ became wide. This signal was assigned to NH−O stretching vibrations and may suggest the interaction of water molecules with both NaDic and HA. Each of them possesses an NH group (Figure 1 and Figure 2) that may be a proton donor and form a hydrogen bond with the carbonyl C=O group, as well as with the −COO^−^ group. The maximum NaDic at 3386 cm^−1^ that was assigned to the NH stretching of the secondary amine disappeared on this spectrum, and this may confirm the interaction between the NH group belonging to NaDic with C=O or the −COO^−^ group of HA. This is consistent with the differences noticed between the spectrum of the physical mixture of F1 components and the spectrum of formulation F1 in the region attributed to COO^−^ group vibrations. These differences are marked in blue in Figure 5. The band of HA at 1604 cm^−1^, related to asymmetrical C=O stretching, and the signal at 1407 cm^−1^, related to the symmetrical C–O stretching of the –COO^−^ group, were absent, confirming the formation of a bond. The NaDic maxima at 1573 cm^−1^ and 1397 cm^−1^, attributed to the symmetric and asymmetric stretching of carboxylate vibrations, changed, indicating the interaction of the COO^−^ groups of NaDic. These differences may therefore suggest the occurrence of a hydrogen bond between the COO^−^ groups of NaDic and the NH group of HA, which was the proton donor.

The interactions between HA and NaDic revealed that formulation F1 may also occur in the hydrogels F2-F5 because both components were present in these preparations. However, the incorporation of the second polymer into the matrix may be responsible for other interactions. The FTIR−ATR spectrum of the physical mixture of the ingredients of formulation F2 with the dried formulation F2 is presented in Figure 6. The strong signal of PA present on the spectrum of the physical mixture at 1702 cm^−1^ was shifted towards 1689 cm^−1^ and its shape and intensity also changed. This peak corresponded to the presence of the C=O group of PA (Figure 10a) and may indicate the formation of a bond with another compound of the formulation [49,64].

The lack of the band of NaDic at 3386 cm^−1^ and the shifting and broadening of the band at 3320 cm^−1^ on the FTIR−ATR spectrum of formulation F2 may suggest the formation of a hydrogen bond of PA with the NH group of the drug. This interaction may also explain the increase in the viscosity of formulation F2 in comparison to the viscosity of formulation F1. Additionally, the bond formation between NaDic and PA correlated well with the release study. Drug release was slowest for formulation F2 containing PA, most likely due to the formation of hydrogen bonds.

The FTIR−ATR spectrum of the F3 physical mixture with the spectrum of formulation F3 is shown in Figure 7. All the characteristic bands of the compounds forming the formulation F3 were observed on the FTIR−ATR spectrum of the F3 physical mixture, although some weak signals of HA (at 1407, 1376, 1032 cm^−1^) and AX (at 1544, 1440, 1388 cm^−1^) were slightly shifted or overlapped by stronger peaks belonging to NaDic. When comparing the spectrum of the physical mixture of F3 with the spectrum of formulation F3, the main discrepancy was noticed in the region marked in blue in Figure 7. The band of AX at 1640 cm^−1^ appeared on the spectrum of formulation F3 at 1692 cm^−1^, and simultaneously the signal of NaDic at 1397 cm^−1^ was shifted towards 1379 cm^−1^. These differences may arise from the formation of hydrogen bonds between amino and/or carboxyl groups of AX with the amino and/or carbonyl groups of the drug. The structure of AX is presented in Figure 10b [65].

The FTIR-ATR spectra of the F4 composition and the physical mixture of the ingredients of F4 are presented in Figure 8. The difference between these spectra concerns the NaDic band shifting from 1397 cm^−1^ towards 1375 cm^−1^, although at this wavenumber there was also a signal from HA. This signal was assigned to the carboxylate group, suggesting the interaction of NaDic with the polymeric component of the matrix. Furthermore, the shift in the NH-related band of NaDic from 3386 cm^−1^ to 3588 cm^−1^, along with the displacement of the C−N stretching band from 1167 cm^−1^ to 1150 cm^−1^ in formulation F4, suggests the formation of hydrogen bonds, where the NH group of NaDic is a proton donor.

The signals of the PVA−PVP mixture at 3434 cm^−1^ and 1654 cm^−1^ were shifted to 3588 cm^−1^ and 1642 cm^−1^, respectively. The structure of PVA and PVP is presented in Figure 10c and 10d, respectively [66]. These compounds possess the C=O group, which may be responsible for the bands shifting. Additionally, the C=O may group be a proton acceptor, meaning that the formation of a hydrogen bond with NaDic is possible. Gull et al. [40] found that the C=O group from PVP interacts with the amino and −OH group from chitosan via hydrogen bonding. It was interesting that the viscosity of formulation F4 was lower than the viscosity of F1, and that the drug was released the fastest from the F4 hydrogel. This may be explained by the fact that the addition of the PVA−PVP mixture to the matrix disrupted the three−dimensional structure of the HA hydrogel, resulting in its partial depolymerization or a change the network arrangement, leading to a decrease in viscosity [51]. The decreased viscosity of F4 accelerated the release of NaDic from the matrix.

The FTIR−ATR spectrum of formulation F5 with the spectrum of its physical mixture is shown in Figure 9. It was noticed that the peak of NaDic at 1397 cm^−1^ appeared on the spectrum of F5 at 1379 cm^−1^. This signal may also be assigned to the band of PEG, which was not observed at 1341 cm^−1^. It also may belong to HA because, on the spectrum of pure HA, a weak signal at 1376 cm^−1^ is present, although it was weaker than the one observed [63]. This region is characteristic for C=O vibrations in the carboxylate and may indicate the interaction of NaDic or HA. PEG does not possess a C=O group in its structure (Figure 10e) [67]. The oxygen atoms in the PEG ether chain can be hydrogen acceptors. It is therefore possible to form hydrogen bonds with the proton donating COOH or NH groups of NaDic.

The broad band on the spectrum of F5 at 3231 cm^−1^ and the lack of a clear peak of NaDic at 3386 cm^−1^ may suggest the formation of a hydrogen bond, in which NaDic is the proton donor. Additionally, the shifting of the NaDic bond at 1167 cm^−1^, caused by C−N stretching, to 1149 cm^−1^ on the spectrum of formulation F5 may also confirm the participation of the NH group in the formation of a bond with the polymer.

The lack of an increase in the viscosity of formulation F5 compared to formulation F1 is noteworthy due to the potential occurrence of hydrogen bonds. The addition of PEG decreased the cross-link density of the hydrogel due to the loosening of the network structure.

It may be concluded that the addition of a second polymer to a carrier based on a natural polymer such as HA may offer improved properties for drug release applications. The possibility of bond formation enables the production of low-cost materials with novel properties. Modifying HA by blending it with other polymers can enhance its practical applications and improve the properties of hydrogels as devices for controlled or targeted drug delivery.

### 2.3. DSC Analysis

**Figure 11:**
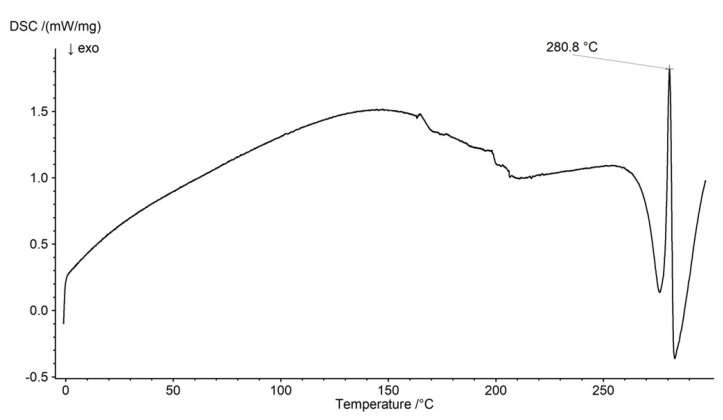
The thermogram of pure NaDic.

The thermogram of pure NaDic is presented in Figure 11. The main endothermic peak was found at 280.8 °C. It correlated well with the signals reported in other research [68,69]. The DSC curves of the polymers used in this study were presented in our previous work [44,63].

**Figure 12:**
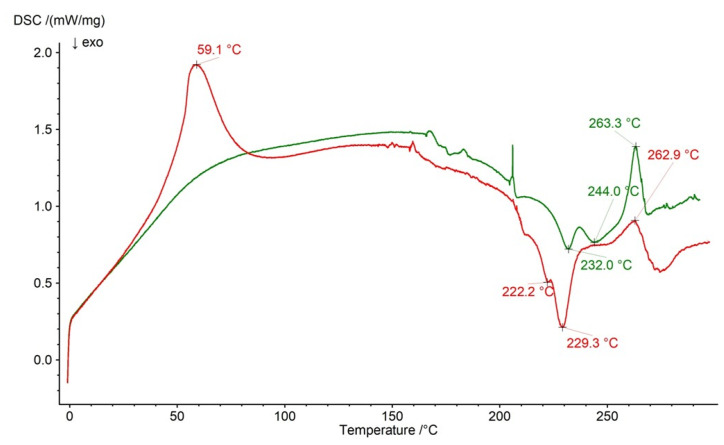
The thermogram of formulation F1 (red) and the physical mixture of its compounds: NaDic, HA (green); arrows indicate the temperatures of exotherms and endotherms.

The thermal profiles of formulation F1 and a physical mixture of its compounds are shown in Figure 12. The endotherm of NaDic was found on the curve of the physical mixture at 263.3 °C and two exotherms of HA were observed at 232.0 and at 244.0 °C. In the case of the DSC profile of formulation F1, the maximum NaDic was present at 262.9 °C, whereas the signals of HA were slightly moved and were found at 222.2 °C and 229.3 °C. These differences between the physical mixture of compounds and formulation F1 may confirm the interaction of HA, at least between the HA chains. The maximum at 59.1 °C present on the thermogram of formulation F1 was the result of water evaporation from the preparation.

**Figure 13:**
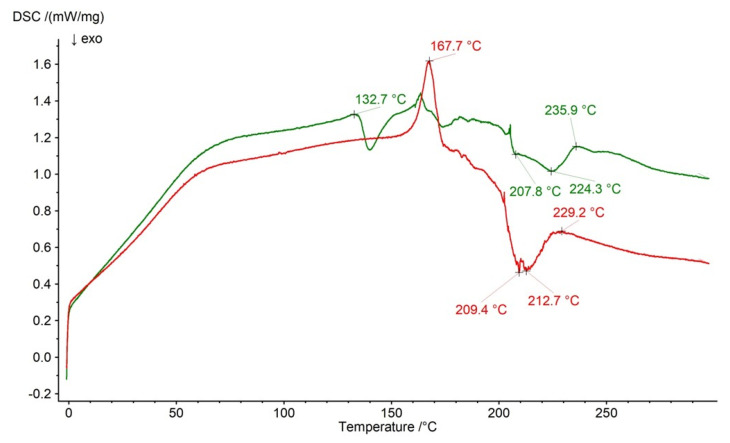
The thermogram of formulation F2 (red) and the physical mixture of its compounds: NaDic, HA, PA (green); arrows indicate the temperatures of exotherms and endotherms.

The DSC curves of formulation F2 and the physical mixture of its compounds are presented in Figure 13. The signals of NaDic were observed on both plots at 229.2 °C and 235.9 °C, respectively. The exotherms of HA were noticed at 207.8 °C and 224.3 °C on the curve of the F2 physical mixture and at 209.4 °C and 212.7 °C on the curve of formulation F2. These signals covered the endotherm of PA that should be observed at 218.1 °C [63]. The weak endotherm at 132.7 °C on the curve of the physical mixture of F2 may be assigned to HA because such signal was observed on the thermal profile of pure HA [63]. It is interesting the appearance of the endotherm at 167.7 °C on the thermogram of formulation F2. It probably belongs to NaDic, although the signal at this temperature on the thermogram of NaDic (Figure 11) was very weak. These differences may confirm the interaction between the drug and the carrier, as proposed in the FTIR study. This interaction may explain the prolonged drug release from all the formulations studied in this work.

**Figure 14:**
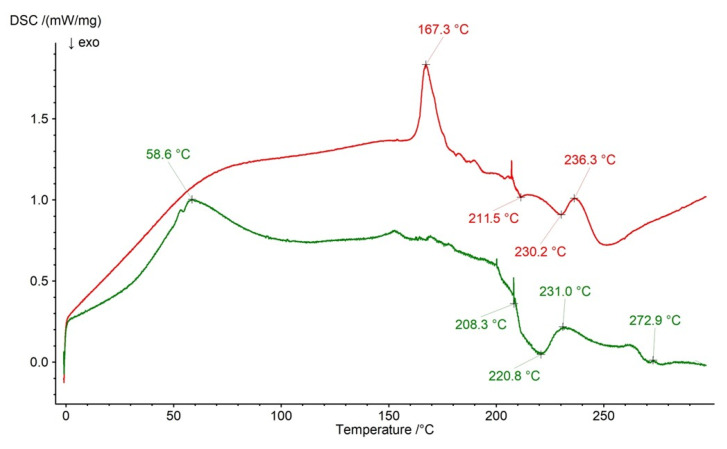
The thermogram of formulation F3 (red) and the physical mixture of its compounds: NaDic, HA, AX (green); arrows indicate the temperatures of exotherms and endotherms.

The thermal behaviors of formulation F3 as well as the physical mixture of F3’s ingredients are shown in Figure 14. The weak signal of NaDic on the F3 curve was found at 272.9 °C, although it was not present on the profile of F3’s physical mixture. Additionally, the new endotherm at 167.3 °C on the F3 curve appeared, similarly to that on the F2 profile, which may also indicate the interaction of the drug with the additional polymer, in this case with AX. Two exotherms of HA were present on the thermogram of F3’s physical mixture at 208.3 °C and 220.8 °C, whereas in the case of the thermal dependence of formulation F3, they were slightly moved to 211.5 °C and 230.2 °C, respectively. The signals of AX on the profile of F3’s physical mixture were present at 58.6 °C and 231.0 °C. However, the endotherm of AX at 58.6 °C was not observed on the thermogram of F3 and the second signal was noticed at 236.3 °C. The remaining signals of pure AX reported in our previous work [44] were difficult to find on the curve of F3’s physical mixture, due to overlapping caused by the peaks of the other components.

**Figure 15:**
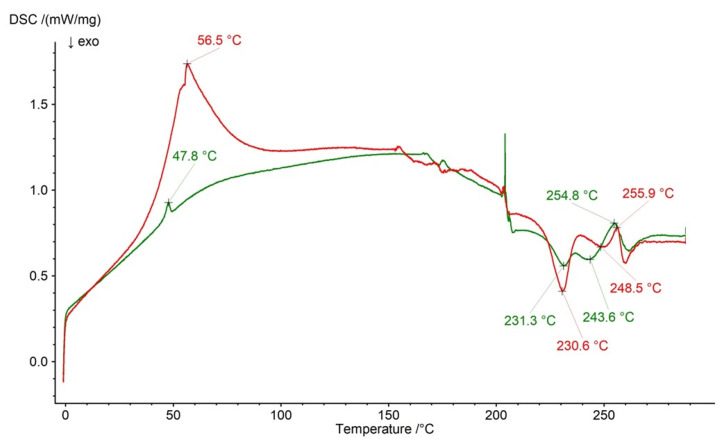
The thermogram of formulation F4 (red) and the physical mixture of its compounds: NaDic, HA, PVA−PVP mixture (green); arrows indicate the temperatures of exotherms and endotherms.

Figure 15 presents the thermograms of formulation F4 and the physical mixture of formulation F4’s compounds. The endotherms of NaDic were found at 255.9 °C and 254.8 °C, respectively. The exotherms of HA at 231.3 °C and 241.9 °C on the curve of F4’s physical mixture appeared at 230.6 °C and 248.5 °C on the thermogram of formulation F4. The signal of the PVA−PVP mixture noticed at 47.8 °C on the thermal profile of F4’s physical mixture occurred at 56.5 °C on the thermogram of formulation F4 and became more clear. This slight change may suggest the interaction of the PVA−PVP mixture with another compound from the formulation postulated in the FTIR−ATR investigations.

**Figure 16:**
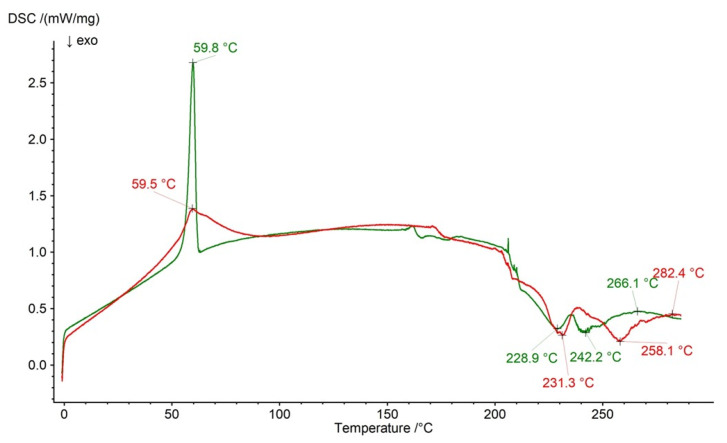
The thermogram of formulation F5 (red) and the physical mixture of its compounds: NaDic, HA, PEG (green); arrows indicate the temperatures of exotherms and endotherms.

The thermogram of the physical mixture of formulation F5’s ingredients and the thermogram of formulation F5 are shown in Figure 16. The endotherm of NaDic was noticed on the DSC curve of F5’s physical mixture at 266.1 °C and at 282.4 on the thermal profile of formulation F5. The exotherms belonging to HA were found at 228.9 °C and 242.2 °C with regard to the thermal dependence of F5’s physical mixture and at 231.3 °C and 258.1 °C on the thermogram of formulation F5. The sharp peak assigned to PEG present on the F5 physical mixture at 59.8 °C was observed on the thermogram of formulation F5 at 59.5 °C, although its shape changed distinctly.

### 2.4. Release Study

**Figure 17:**
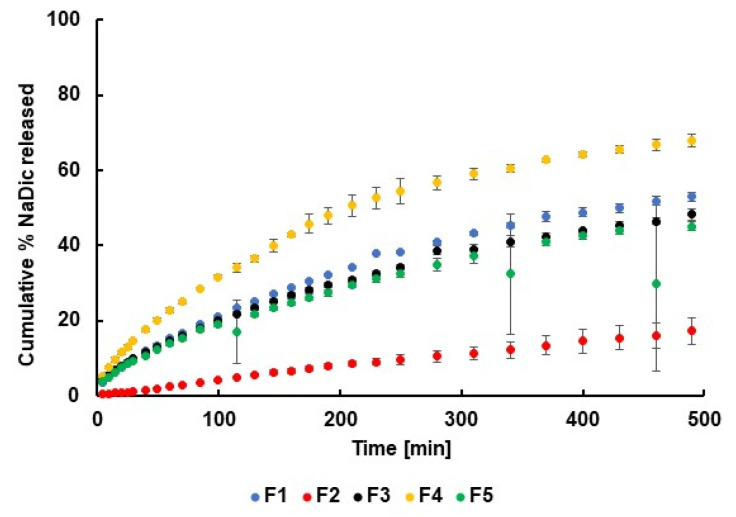
The release curves of NaDic from formulations F1−F5, *n* = 6; black markers indicate error bars.

The drug dissolution study was performed using PBS at pH 6.8 as the acceptor fluid, reflecting the pH of inflamed synovial fluid; normal synovial fluid has a pH of 7.2–7.4 [70,71,72,73]. The NaDic release curves obtained from formulations F1−F5 are presented in Figure 17.

It was observed that none of the hydrogels released 100% of the drug within 8 h. The lowest amount of NaDic was released from formulation F2, which was doped with PA. This finding is consistent with the viscosity study, which indicated that the dynamic viscosity of F2 was higher than that of the other formulations. In hydrogels with higher viscosity, the movement of drug molecules within the densely packed polymer network is more limited compared to formulations with lower viscosity. This resulted in slower drug release due to hindered diffusion. Moreover, higher viscosity is associated with increased polymer cross-linking, which reduces pore size and further restricts drug transport [74,75,76,77]. The highest amount of drug was released from formulation F4, which had a lower dynamic viscosity than F2, supporting the explanation above. The lowest dynamic viscosity was observed in formulation F5; however, the amount of drug released from this hydrogel was similar to that released from F1 and F3. This may be due to interactions between the drug and the carrier. Additionally, while viscosity determines flow resistance, it does not fully define the hydrogel’s microstructure. Therefore, a hydrogel with low viscosity but a tightly packed structure can still reduce the drug release [74,75]. Differences between the release curves were analyzed by calculating the difference f_1_ and similarity f_2_ factors, and the results are presented in Table 3.

According to FDA recommendations [78,79], dissolution profiles are considered similar when the f_1_ value is below 15 and the f_2_ value is above 50. This indicates discrepancies between the drug dissolution profiles of F1 and F2, as well as between F1 and F4. Statistical analysis using Student’s *t*-test further confirmed significant differences between the dissolution profiles of F1 vs. F2 and F1 vs. F4, suggesting that the addition of PA or the PVA–PVP mixture influenced NaDic transport from the matrix to the acceptor fluid.

Mikusova et al. [75] studied the influence of gel-forming polymers and their structure on the rheological properties of hydrogels and drug release. It was also found that incorporating a drug into the hydrogel matrix can affect its viscosity. In some cases, the presence of the drug increased the stiffness of the polymer network. Drug transport from the hydrogel may also depend on the charge of the polymer chains. The stabilization of drug–polymer associations by Coulombic interactions may explain the slower drug release. In the present study, NaDic was released the slowest from hydrogel F2, which was composed of HA and PA. Both of these ingredients are polyanions; therefore, electrostatic repulsion between these macromolecules would be expected, potentially leading to reduced viscosity and an increased drug release rate compared to formulation F1, which did not contain a second polymer. However, the opposite effect was observed, suggesting that other types of interactions may dominate.

Djekic et al. [77] reported that a large number of carboxyl groups in the carbomer structure allows for the formation of hydrogen bonds with proton-acceptor groups of substrates. In our previous work [44], interactions were found between the –NH group of HA and the –COO^−^ group of PA, as well as between the –C=O group of HA and the –COO^−^ group of PA.

It can be concluded that the addition of various polymers to the HA-based hydrogel modified its structure, which in turn affected the drug release profile.

### 2.5. Kinetic Analysis

The obtained release profiles were analyzed using several kinetic models such as zero-, first-, second-order kinetics, as well as Higuchi, Korsmeyer−Peppas and Peppas−Sahlin models. The zero-order equation corresponds to a constant drug release, where the same amount of drug is transferred from the matrix to the medium at equal time intervals. This relationship is exploited in drug delivery systems such as transdermal slow-release matrices. First-order kinetics describe dissolution that depends on the drug concentration; the percentage of drug released remains constant over equal time intervals. In a second-order kinetic model, the release rate of an active substance is proportional to the square of its concentration remaining in the matrix. In drug delivery systems, it describes profiles in which a large amount of drug is initially released, but over time, the release rate rapidly decreases as the concentration remains nonlinearly low. Systems with second-order kinetics are less common than zero- or first-order kinetics, but they are a valuable tool in the design of formulations in which simultaneous chemical reactions determine the drug release profile [80,81]. The Higuchi equation is used to characterize the drug release kinetics driven by diffusion. According to this, the amount of active substance released is proportional to the square root of time. It is a particular case of the Korsmeyer−Peppas model [74,76]. The Korsmeyer–Peppas model is widely applied to describe drug release from hydrogels. This semi-empirical model relates drug release to the elapsed time through an exponential equation. It is particularly informative due to the presence of parameter *n* in Equation (7), which reflects different drug release mechanisms [82]. According to this model, the parameter *n* can be interpreted based on the solvent diffusion rate (R_diff_) and polymer chain relaxation rate (R_relax_). For drug release from film, an *n* value below 0.5 indicates diffusion-related release (Case−I diffusion) and is followed by Fick’s law. The rate of solvent penetration is smaller than the rate of polymer chain relaxation (R_diff_ < < R_relax_). Values of *n* that range from 0.5 to 1.0 describe anomalous (non-Fickian) transport where the diffusion and relaxation rates are similar (R_diff_ ≈ R_relax_). The release is considered a hybrid release mechanism. The value of *n* = 1.0 indicates Case II transport (zero-order release) and describes a relaxation-controlled process, where diffusion is much faster than polymer relaxation (R_diff_ > > R_relax_). It is also possible that the drug release is ruled by both the diffusion and relaxation of the polymer molecule. In this case, the value of *n* is above 1.0 and the mechanism is called super Case−II transport [76,83].

The Peppas–Sahlin equation allows one to estimate the contributions of Fickian diffusion and polymer relaxation mechanisms to an anomalous release process [83].

**Figure 18:**
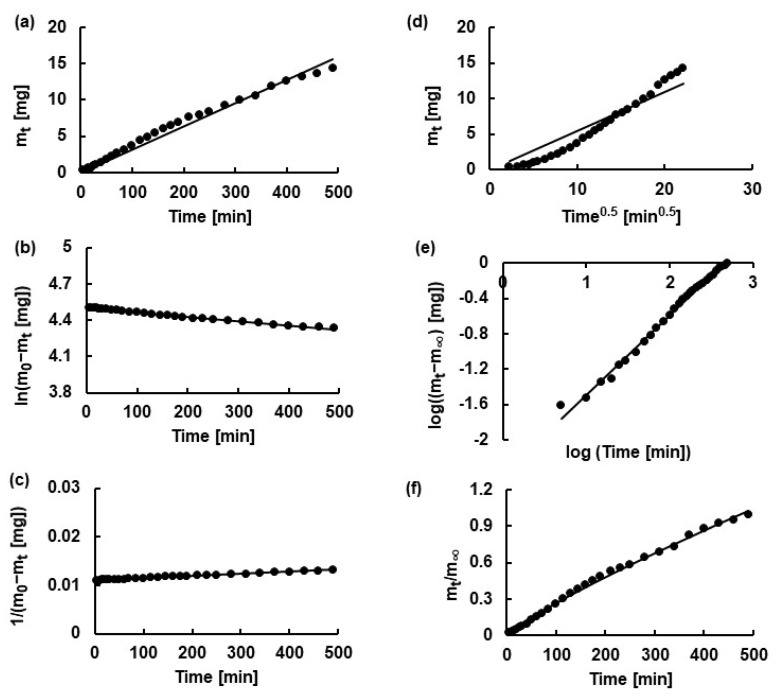
Examples of fitting the theoretical lines (—) to the experimental points (•) obtained from NaDic release from formulation F2, based on (**a**) zero-order, (**b**) first-order, (**c**) second-order, (**d**) Higuchi (**e**). Korsmeyer−Peppas, and (**f**) Peppas−Sahlin equations.

The fitting of the experimental data to theoretical curves is presented in Figure 18. From this analysis, kinetic parameters such as release rate constants, the half-release time, diffusional exponents, and the coefficients of determination were derived. The calculated values of these parameters are listed in Table 4. It should be noted that, for most datasets, more than one model was able to describe the dissolution process, as indicated by R^2^ values close to 1, with only minor differences between some models. Among all the formulations studied, the second-order kinetics and the Korsmeyer–Peppas model provided the best fit, with R^2^ values ranging from 0.9908 ± 0.0088 to 0.9971 ± 0.0032 for the second-order model, and from 0.9933 ± 0.0023 to 0.9983 ± 0.0005 for the Korsmeyer–Peppas model.

The values of n derived from the Korsmeyer–Peppas equation range from 0.56 ± 0.01 to 0.88 ± 0.04, indicating that NaDic release from formulations F1−F5 follows anomalous (non-Fickian) transport. This means that the release is controlled by polymer relaxation or the swelling of the matrix.

The first term on the right-hand side of Equation (8), ($k_{1P-S}t^{n’}$), represents the Fickian contribution, while the second term characterizes the Case II relaxational contribution. It should be noted that the value of k_1P-S_ is four orders of magnitude lower than the value of k_2P-S_, confirming that drug release occurs predominantly via non-Fickian transport. It may be assumed that the value of k_1P-S_ is practically close to 0.

The release rate constants obtained from the applied equations varied. However, based on all the models used, the release rate constant of NaDic from formulation F2 was the lowest, while the highest release rate constant was observed for formulation F4. This is consistent with the release profiles, in which the highest amount of drug released within 8 h was released from hydrogel F4; meanwhile, the lowest concentration of NaDic was noticed in the acceptor fluid of F2.

**Figure 19:**
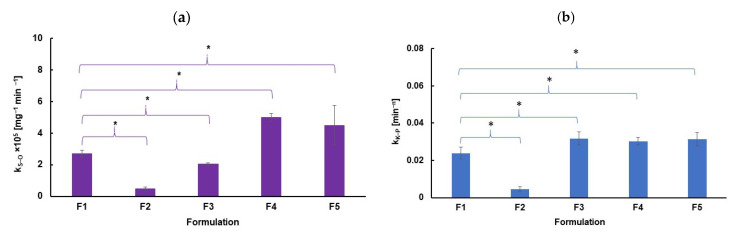
The comparison of the release rate constants obtained from (**a**) second-order kinetics (purple) and (**b**) Korsmeyer−Peppas equation (blue); F1 was established as the reference sample. The bars with asterisks (*) indicate a statistically significant difference, *n* = 6; *p* = 0.05.

It was mentioned that the second-order kinetics and Korsmeyer–Peppas equation were the most appropriate models for describing the release of NaDic from formulations F1−F5. The comparison of the calculated release rate constants, k_S−O_ and k_K−P_, is presented in Figure 19. Statistical analysis based on Student’s *t*-test, using formulation F1 as the reference, indicated significant differences between the release rate constants of F1 and those of the other formulations. These differences were associated with the addition of an auxiliary polymer to formulations F2−F5. The observed variations in the release rate constants result from doping the hydrogels with synthetic polymers, which is consistent with the viscosity study results. The viscosities of formulations F2−F5, doped with synthetic polymers, differed from that of F1, which is based solely on HA.

It is interesting that differences in the release rate constants, k_S−O_ and k_K−P_, between F1 and the other formulations were observed, even though the release profiles of F1, F3, and F5 were found to be similar.

The differences observed in the release rate constants k_S−O_ and k_K−P_ between formulation F1 and the other systems were evaluated using Student’s *t*-test at a 95% confidence level, which is a standard approach in such analyses. However, increasing the confidence level—for example, from 95% to 99%—makes it more difficult to demonstrate statistical significance. The confidence level reflects how certain the result is; a 95% level implies accepting a 5% risk of a Type I error (i.e., incorrectly rejecting the null hypothesis), whereas a 99% confidence level reduces this risk to 1%. As a result, the corresponding critical value of t becomes higher, and the observed differences must be more pronounced (i.e., larger effect size or smaller variance) to achieve significance. Therefore, differences that appear statistically significant at the 95% level may no longer meet the threshold at 99%, suggesting that such findings should be interpreted with caution [84,85].

To assess the similarity of the release profiles themselves, we used f_1_ (difference factor) and f_2_ (similarity factor), which are recommended by both the FDA and EMA for comparing dissolution profiles and are considered more reliable and robust in current pharmaceutical practice [78].

Although the release rate constants indicated some statistically significant differences among formulations, the f_1_/f_2_ analysis showed that the release profiles of F1, F3, and F5 were similar. It is possible that higher concentrations of the additional polymer would result in more distinguishable release profiles, but at the levels used in these formulations, such differences were not evident.

In contrast, formulation F2 (containing PA) was the only system that demonstrated both statistically significant differences in its release rate constants and a distinct release profile compared to F1. Moreover, F2 showed the lowest cumulative NaDic release over the 8 h test period. These findings lead to the conclusion that F2 is the most promising formulation for achieving prolonged drug release.

The obtained results allow us to state that the most influential factor in F1–F5 is the formation of hydrogen bonds between NaDic and the polymer components. These interactions play a key role in modulating drug release, which is the central focus of our study. Hydrogen bonding not only affects the rate and profile of NaDic release, but also contributes significantly to the viscosity of the hydrogel systems. This, in turn, may determine the injectability of the formulation, as an appropriate viscosity is essential for administration through a syringe into the joint cavity.

While other factors such as polymer charge or electrostatic repulsion may also be present, their role appears secondary in comparison to the impact of hydrogen bonding observed in our systems. Therefore, we consider hydrogen bond formation to be the most critical interaction governing both the physicochemical properties and therapeutic performance of the formulations.

## 3. Materials and Methods

Diclofenac sodium (NaDic) was obtained from POL−AURA (batch 646SEA, Dywity, Poland), and high-molecular-weight sodium hyaluronate (HA) with molecular weight > 1.10 MDa was purchased from ESCENT (batch 20231012, Szczecin, Poland). Polyacrylic acid (Carbopol, PA) was bought from LUBRIZOL (lot no. 0102457428, Wickliffe, OH, USA), ammonium acryloyldimethyltaurate/VP copolymer (Aristoflex AVC, AX) came from CLARIANT INTERNATIONAL Ltd. (batch 13824026892, Muttenz, Switzerland), the polyvinyl acetate–polyvinylpyrrolidone mixture (PVA−PVP, Kollidon SR) was delivered from BASF (batch 16090424U0, Ludwigshafen, Germany) and polyethylene glycol 4000 (PEG) was obtained from POL−AURA (batch CHJ594, Morąg, Poland). Trisodium phosphate dodecahydrate was bought from CHEMPUR (serial no. 16/03/01, Piekary Śląskie, Poland) and hydrochloric acid 35−38% was provided by AVANTOR PERFORMANCE MATERIALS (serial no. 1023/12/13, Piekary Śląskie, Poland). All chemicals were used as supplied, without further purification. Semi-permeable cellulose membranes with a retention range of 5−8 μm were obtained from Carl Roth (lot no. 13−155, Karlsruhe, Germany).

### 3.1. Hydrogels Preparation

The procedure for obtaining the hydrogels was the same as in our previous study [44]. Briefly, HA or HA and one of the synthetic polymers (PA or AX or PVA−PVP or PEG) was/were introduced into the mortar, followed by the addition of NaDic. The ingredients were ground manually and then were brought to a homogeneous form using a homogenizer (Unidrive X 1000D, Cat, Staufen, Germany). The rotation speed was set at 16,000 rpm and the homogenization process was carried out for 10 min. The hydrogel was stored at 6 °C for 24 h. After this time, pH and viscosity measurements were performed, along with drug release testing. The composition of the hydrogels is presented in Table 1.

The concentration of the drug in the obtained formulations was 1% (*w*/*w*), the concentration of HA was 2% (*w*/*w*), and the concentration of the synthetic polymer was 0.5% (*w*/*w*). Commercially available NaDic-based hydrogels contain the drug at concentrations of 10 mg/g, 20 mg/g, or 30 mg/g, corresponding to 1% (*w*/*w*), 2% (*w*/*w*), or 3% (*w*/*w*), respectively [86,87,88]. The HA-based intra-articular hydrogels available on the market contain 16.0 mg of HA in 1 mL (1.6%), 22.0 mg in 1 mL (2.2%), or 25.0 mg in 2 mL (2.5%) [89,90,91]. The concentrations of the synthetic polymers used in formulations F2–F5 were the same as those applied in our previous work [44].

### 3.2. pH and Viscosity Measurements

pH measurements were conducted at room temperature using a pH meter (CX-601, ELMETRON, Zabrze, Poland) connected to a dedicated electrode (HYDROMET ERH-11S, Gliwice, Poland). The pH of each formulation was measured six times, and the mean value was calculated.

Viscosity measurements of the hydrogels were performed at both room temperature and 37 °C. The viscosity study was carried out using a rotational viscometer (DV2T, Brookfield, Middleboro, MA, USA) at a rotation speed of 50 rpm, employing spindle No. 4, 5, or 6 depending on the formulation. The dynamic viscosity was measured six times for each sample, and the mean value was calculated.

### 3.3. ATR−FTIR Measurements

The obtained hydrogels were dried for approximately two weeks at 6 °C, and then crushed in a mortar. ATR–FTIR (attenuated total reflectance–Fourier transform infrared) measurements were performed using an FTIR spectrometer in ATR mode (Nicolet iS50, Thermo Scientific, Waltham, MA, USA). Thirty-two scans per sample were collected at a resolution of 4 cm^−1^ over the range of 4000–400 cm^−1^, with a scanning speed of 65 scans per minute at room temperature. Background measurements were performed before each sample measurement. All dried formulations, physical mixtures of formulation ingredients, and pure hydrogel components were analyzed.

### 3.4. DSC Study

The dried preparations for F1–F5 were subjected to calorimetric analysis using a differential scanning calorimeter (DSC 214 Polyma, Netzsch, Selb, Germany). Physical mixtures, consisting of the components of the corresponding formulations F1–F5, as well as the pure ingredients present in the preparations, were also analyzed. Samples of 3–5 mg were placed in aluminum crucibles covered with pierced lids and lightly pressed. An empty pan, sealed in the same manner as the samples, was used as a reference. Measurements were carried out over the temperature range of 0–300 °C at a heating rate of 5 °C/min under a nitrogen atmosphere with a flow rate of 25 mL/min.

### 3.5. Release Tests

The release of NaDic from formulations F1–F5 was studied using the paddle-over-extraction-cell apparatus in accordance with Ph. Eur. 11.0 [92]. Hydrogel samples were introduced into six disks, which were placed in six chambers containing 1 L of acceptor fluid. The release medium was phosphate-buffered saline (PBS) at pH 6.8, prepared according to FP XII [93]. Dissolution testing was performed using dedicated equipment (ERWEKA DT 126 Light, Heusenstamm, Germany) with a paddle rotation speed of 50 rpm at a temperature of 37 °C. At predefined time intervals, 3 mL samples of the acceptor medium were withdrawn and replaced with an equal volume of fresh PBS. The concentration of NaDic in the collected samples was determined spectrophotometrically using a UV-Vis spectrophotometer (JASCO V-530, Tokyo, Japan). The spectrum of NaDic was recorded, and the calibration curve was constructed at the maximum absorption wavelength of 276 nm, where NaDic exhibited the highest absorbance and other formulation components did not interfere [44]. The obtained absorbance values were converted to the amount of released drug based on the calibration curve.

### 3.6. Difference Factor f_1_ and Similarity Factor f_2_

A comparison of the dissolution profiles was performed by determining the difference factor f_1_ and the similarity factor f_2_ from Equations (3)−(4) presented below:

(3) $$f_{1}=\frac{\sum_{t=1}^{n}\left|R_{t}-T_{t}\right|}{\sum_{t=1}^{n}R_{t}}\times 100$$

(4) $$f_{2}=50\times log\left\{{\left[1+\frac{\sum_{t=1}^{n}{\left(R_{t}-T_{t}\right)}^{2}}{n}\right]}^{-0.5}\times 100\right\}$$

where n is the number of time points, R_t_ is the released value of the reference batch at time t, and T_t_ is the released value of the test batch at time t.

According to FDA guidelines, release profiles are considered to be the same if the value of f_1_ is between 0 and 15 and, simultaneously, if the f_2_ coefficient value is in the range of 50−100. The f_1_ value measures the percent difference between two profiles at each time point and indicates dissimilarity (values between 0 and 15 are generally considered acceptable). The f_2_ value measures the similarity between two profiles, with values between 50 and 100 indicating that the profiles are similar.

These parameters are commonly used to assess how closely a test formulation matches a reference in terms of drug release behavior. We believe this addition will help non-specialist readers better understand the relevance of these values in the context of our study [78,94,95].

### 3.7. Kinetic Calculations

The dissolution profiles were analyzed by employing zero-, first-, second-order kinetics, as well as Higuchi, Korsmeyer–Peppas and Peppas–Sahlin models according to the following equations:

(5) $$\text{zero-order}\;\;\;m_{t}=m_{b}+k_{0}t$$

(6) $$\text{first-order}\;\;\;\ln\left(m_{0}-m_{t}\right)=\ln\left(m_{0}\right)-k_{1}t$$

(7) $$\text{second-order}\;\;\;\frac{1}{\left(m_{0}-m_{t}\right)}=\frac{1}{m_{0}}-k_{2}t$$

(8) $$\mathrm{Higuchi}\;\;\;m_{t}=k_{H}t^{0.5}$$

(9) $$\mathrm{Korsmeyer}–\mathrm{Peppas}\;\;\;\log\left(\frac{m_{t}}{m_{\infty }}\right)=\log k_{K-P}+n\log t$$

(10) $$\mathrm{Peppas}–\mathrm{Sahlin}\;\;\;\frac{m_{t}}{m_{\infty }}=k_{1P-S}t^{n’}+k_{2P-S}t^{2n’}$$

where m_t_ is the amount of the drug released in time t; m_b_ is the amount of the drug in the solution before the release, which is usually 0; k_0_ is the zero-order release rate constant; m_0_ is the amount of the drug in the formulation before the dissolution; k_1_ is the first-order release rate constant; k_2_ is the second-order release rate constant; k_H_ is the Higuchi release rate constant; m_∞_ is the amount of the drug released after infinitive time, k_K-P_ is the Korsmeyer–Peppas release rate constant, n is the parameter indicative of the drug release mechanism; k_1P-S_ is the Peppas–Sahlin release rate constant for the Fickian contribution to drug release, k_2P-S_ is the Peppas–Sahlin release rate constant for the Case II contribution of the drug release, and n’ is the diffusional exponent from the Peppas–Sahlin equation [44,83,96,97,98].

Based on Equations (5)−(10), the kinetic parameters, including the release rate constants, half-release time and parameters n and n’, were calculated.

### 3.8. Statistical Analysis

The calculated mean values of the parameters were obtained as the arithmetic mean of six measurements and are presented together with the standard deviation (SD). Differences in the viscosity of formulations F1–F5 were analyzed using the F-test and Student’s *t*-test at a 95% confidence level. The effect of incorporating the second polymer into formulations F2–F5 on the release of NaDic was also evaluated using the F-test and Student’s *t*-test at the 95% significance level. This analysis enabled the assessment of the significance of differences between experimental groups at each time point [74]. Formulation F1, which did not contain a synthetic polymer, was used as the reference preparation. Kinetic parameters were derived using the least squares method. Based on this analysis, the correlation coefficient (R^2^) was also determined.

## 4. Conclusions

This study revealed that the incorporation of NaDic into the hydrogels influenced the dynamic viscosity of the preparations. The differences arose from the electrostatic repulsions between the polymer chains and the drug. Additionally, hydrogen bonds were formed between NaDic and the carrier. These interactions affect the release of the drug from the hydrogels. The drug was released from the formulations via anomalous (non-Fickian) transport. The second-order kinetics and Korsmeyer−Peppas model most convincingly characterized the dissolution process. The formulation based on HA doped with PA was proposed as the formulation that may efficiently prolong drug release to the acceptor environment.

Understanding the viscometrical properties, structure and interactions of hydrogels, as well as their drug release kinetics, may enable the development of a variety of formulations with modified drug release profiles tailored to specific drug delivery applications.

## Figures and Tables

**Table 1:** The composition of the formulations studied.

Formulation	NaDic [g]	HA [g]	PA [g]	AX [g]	PVA− PVP [g]	PEG [g]	Water [g]
F1	2	4	—	—	—	—	194
F2	2	4	1	—	—	—	193
F3	2	4	—	1	—	—	193
F4	2	4	—	—	1	—	193
F5	2	4	—	—	—	1	193

**Table 2:** pH of formulations F1-F5 at room temperature.

Formulation	F1	F2	F3	F4	F5
pH	8.20 ± 0.11	5.71 ± 0.05	7.87 ± 0.03	7.71 ± 0.01	7.69 ± 0.05

**Table 3:** The values of difference f_1_ and similarity factors f_2_, obtained from the comparison of the dissolution profiles of NaDic from formulations F1−F5.

	f_1_				f_2_			
Formulation	F2	F3	F4	F5	F2	F3	F4	F5
F1	75.06	8.33	39.97	15.96	31.74	75.20	45.88	59.91

**Table 4:** The calculated kinetic parameters of the NaDic release from hydrogels F1−F5.

Kinetic Model	Kinetic Parameters	F1	F2	F3	F4	F5
Z−O	k_0_ × 10 [mg × min^−1^]	1.24 ± 0.10	0.34 ± 0.01	1.08 ± 0.09	1.57 ± 0.16	0.89 ± 0.10
	t_0.5_ [min]	363 ± 29	1371 ± 43	413 ± 36	277 ± 28	2460 ± 58
	R^2^	0.9571 ± 0.0049	0.9932 ± 0.0032	0.9498 ± 0.0044	0.9327 ± 0.0033	0.8962 ± 0.0667
F−O	k_1_ × 10^3^ [min^−1^]	1.59 ± 0.07	0.386 ± 0.013	1.27 ± 0.07	2.31 ± 0.16	1.09 ± 0.17
	t_0.5_ [min]	435 ± 20	1862 ± 65	545 ± 29	301 ± 21	641 ± 115
	R^2^	0.9855 ± 0.0043	0.9922 ± 0.0022	0.9806 ± 0.0056	0.9667 ± 0.0114	0.8176 ± 0.1898
S−O	k_2_ × 10^5^ [mg^−1^ × min^−1^]	2.72 ± 5.12	0.472 ± 0.015	2.03 ± 0.06	5.01 ± 0.16	4.50 ± 0.15
	t_0.5_ [min]	409 ± 8	2461 ± 74	550 ± 16	230 ± 7	306 ± 10
	R^2^	0.9971 ± 0.0032	0.9935 ± 0.0029	0.9939 ± 0.0045	0.9918 ± 0.0091	0.9908 ± 0.0088
H	k_H_ [mg × min^−1/2^]	2.20 ± 0.06	0.568 ± 0.047	1.93 ± 0.04	2.81 ± 0.06	1.59 ± 0.12
	t_0.5_ [min]	421 ± 22	6604 ± 1027	543 ± 20	239 ± 11	663 ± 98
	R^2^	0.9954 ± 0.0015	0.9567 ± 0.016	0.9976 ± 0.0009	0.9963 ± 0.0016	0.9529 ± 0.0502
K−P	k_K-P_ × 10^2^ [min^−n^]	2.39 ± 0.11	0.460 ± 0.001	3.17 ± 0.01	3.38 ± 0.28	3.15 ± 0.20
	t_0.5_ [min]	144 ± 11	224 ± 58	137 ± 13	121 ± 19	131 ± 30
	R^2^	0.9983 ± 0.0005	0.9858 ± 0.0056	0.9971 ± 0.0010	0.9933 ± 0.0023	0.9954 ± 0.0057
	*n*	0.61 ± 0.01	0.88 ± 0.04	0.56 ± 0.01	0.59 ± 0.02	0.56 ± 0.01
P−S	k_1 P-S_ × 10^6^ [min^−n’^]	1.67 ± 4.11	0.62 ± 1.50	0.01 ± 0.001	0.01 ± 0.001	0.01 ± 0.001
	k_2P-S_ × 10^2^ [min^−n’^]	2.68 ± 0.31	0.529 ± 0.19	2.77 ± 1.09	4.25 ± 0.12	3.07 ± 0.27
	n’	0.30 ± 0.01	0.43 ± 0.05	0.30 ± 0.05	0.26 ± 0.26	0.29 ± 0.51
best fit *		S-O, H, **K-P**	Z-O, F-O, **S-O**	S-O, **H**, K-P	S-O, **H**, K-P	S-O, **K-P**

Z−O-zero-order model; F−O-first-order model, S−O- second-order model; H-Higuchi model; K−P-Korsmeyer−Peppas model, P−S-Peppas−Sahlin model. * there was more than one model, which described the release with a high R^2^ value; thus, more than one model is presented in the row. However, the models prevailing in all cases were discussed further in order to compare the data, but the models with the highest R^2^ are marked in bold.

## Data Availability

The data that support the findings of this study are available from the corresponding author, W.M., upon reasonable request.
